# The Influence of Laser Remelting Parameters on the Surface Quality of Copper

**DOI:** 10.3390/mi15070927

**Published:** 2024-07-20

**Authors:** Hengzheng Li, Yang Chen, Shuai Chen, Yan Liu, Conghu Liu

**Affiliations:** 1 School of Mechanical and Electronic Engineering, Suzhou University, Suzhou 234000, China; lihengzheng@ustc.edu.cn (H.L.); melissa1207@126.com (Y.L.); liuconghu@sjtu.edu.cn (C.L.); 2 School of Chemistry and Materials Science, University of Science and Technology of China, Hefei 230026, China

**Keywords:** laser surface remelting, process parameters, surface morphology, surface roughness, wear resistance

## Abstract

In order to improve the surface quality of copper after laser remelting, this article took laser frequency, pulse width, and energy density as the research objects and used scanning electron microscopy (SEM), a laser confocal three-dimensional measurement instrument, hardness tester, and friction and wear measurement instrument to study the surface morphology, surface roughness, microhardness, and wear resistance of copper, respectively. The results indicate that the frequency, pulse width, and energy density of laser remelting could directly affect the surface quality of the sample, but the influence of frequency and pulse width was more significant. When the laser remelting frequency was 10 Hz, the pulse width was 10 ms, and the energy density was 132.69 J/mm^2^, the sample exhibited good surface morphology, roughness, and wear resistance. The relevant research in this article can provide a good reference for the laser surface treatment of copper-based materials.

## 1. Introduction

The surface quality and mechanical properties of materials are important factors determining the performance of components. How to obtain high-quality components under low energy consumption conditions is an important challenge in sustainable manufacturing processes [1,2]. There are two common methods to improve the service life of components. One is to improve the lubrication conditions of the components, and the other is to enhance the surface properties of the materials. In the first method, adding lubricating oil is a common practice. In precision equipment, constructing functional microstructures on the surface of components is also an important method [3,4,5,6]. Functional microstructures can improve the forming quality of the contact surface oil film and the lubrication conditions, thus extending the service life of components. Another approach is to improve the surface performance of components. Common methods include preparing protective coatings, surface nitriding, surface alloying, and laser surface strengthening [7,8,9,10,11,12]. Copper has good conductivity, thermal conductivity, and processability, and is widely used in electronic components, aerospace, automotive industry, and other fields. Due to the low hardness of copper itself, it is prone to wear and tear during use, which greatly limits its application in the engineering field [13,14].

Laser surface strengthening is the use of high-energy laser beams to irradiate material surface, utilizing the interaction between the laser beam and the material to generate beneficial residual stresses on the material surface and achieve the goal of improving the mechanical properties [15,16,17,18,19]. The use of laser technology for the surface strengthening of copper and copper alloys can effectively improve their surface properties. Wei et al. used laser shock peening to treat the surface of pure copper, and research has shown that the overlap percentage of laser shock plays a key role in improving the surface mechanical properties [20]. Wang et al. conducted dynamic simulation and numerical calculation of plastic deformation and residual stress fields during laser strengthening using finite element simulation software [21,22]. Liu et al. prepared a Ni-based silicide coating on the surface of pure copper using laser cladding technology, and analyzed and evaluated the microstructure, wear resistance, and microhardness of the coating under different compositions [23]. Lu et al. systematically studied the effects of coating and temperature on the tensile properties and surface microstructure of H62 brass during laser strengthening, revealing the potential mechanisms of the surface nanocrystallization and amorphization of brass [24]. Park et al. studied the effect of laser energy density on the degradation of uncoated copper contacts. Research has shown that after high-energy density laser shock, the surface hardness of copper can be increased from 55 Hv to 120 Hv, significantly improving the service life of copper contacts [25]. Zhu conducted laser strengthening on the surface of SiCp/Cu composite materials and studied the effects of laser strengthening on the surface microhardness, residual stress, tensile strength, and elongation after fracture of copper-based composites [26]. Gilev et al. conducted laser quenching on the surface of a steel-copper powder pseudo alloy and analyzed the surface microstructure and microhardness of the samples under different parameters. Research has shown that the surface performance of the treated sample is not only dependent on laser parameters, but also on the surface geometry of the sample [11]. Feng used a nanosecond laser to strengthen copper blocks and analyzed the effect of absorption layers composed of different materials on the surface micro defects [27].

Laser surface remelting technology is one of the important components of laser surface strengthening technology [28,29]. This method undergoes a rapid melting and solidification process on the surface of the material during processing, and the bonding between the remelt layer and the material substrate is stable [30]. Laser surface remelting technology can effectively improve the hardness, wear resistance, and corrosion resistance of material surfaces while ensuring the small deformation of components [31,32]. In order to obtain the relationship between the laser remelting parameters and the surface quality and wear resistance of the sample, this paper studied the influence of laser frequency, pulse width, and energy density on the surface morphology, roughness, microhardness and wear resistance of the sample. The influence of process parameters on the surface quality of the sample was analyzed and studied. The relevant research in this article can provide a reference for the laser surface treatment of copper-based materials.

## 2. Experimental

### 2.1. Experimental Materials

Red copper (purity grade of industrial pure) was used as the metal substrate in the experiment, and the sample size was 50 mm × 30 mm × 2 mm. Before the experiment, the sample was cleaned with detergent to remove oil stains on its surface. Then, the sample was polished to remove the oxide layer on its surface. Finally, it was rinsed with deionized water and dried for use.

### 2.2. Laser Equipment and Sample Characterization

**Figure 1:**
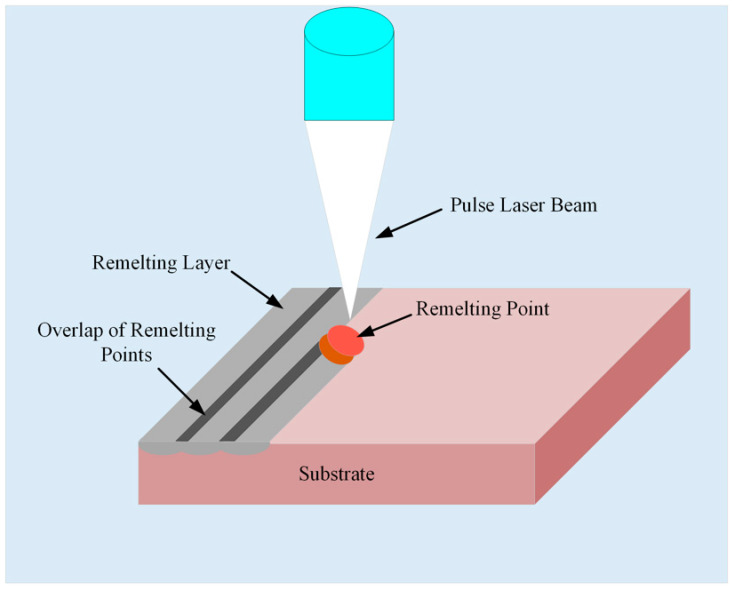
Schematic diagram of the laser melting process.

The sample was surface treated using pulsed laser equipment (TY-LFS-500, Wuhan Tianyi Laser Equipment Co. Ltd., Wuhan, China). The maximum power of this equipment is 14 kW, the working voltage is 380 V, the laser wavelength is 1064 nm, the feed speed of the equipment is 5 mm/min, and the distance between the scanning lines is 0.5 mm. The schematic diagram of the laser melting process is shown in Figure 1.

The surface morphology of the sample was characterized by scanning electron microscopy (SEM, S4800, Hitachi, Japan) with an acceleration voltage of 20 kV. A laser confocal three-dimensional measurement instrument (SM-5100, Sixian Optoelectronics Technology (Shanghai) Co. Ltd., Shanghai, China) was used to model the 3D morphology of the sample surface and measure its surface roughness. The surface roughness measurement results were taken as the average of three measurements. We used a high-speed reciprocating friction and wear testing machine (HSR-2M, Lanzhou Zhongke Kaihua Technology Development Co. Ltd., Lanzhou, China) to conduct wear tests on the samples. A GCr15 alloy ball with a diameter of 4 mm was used for wear with a frictional load of 100 g and a friction time of 5 min. The reciprocating friction stroke was 5 mm, and the reciprocating frequency was 500 times/min. The microhardness of the sample surface was tested using a microhardness tester (HVS-1000Z, Shanghai Optical Instrument Fifth Factory Co. Ltd., Shanghai, China). The test load was 50 g and the loading time was 15 s. We then randomly tested five points on the surface of the sample, and took the average as the microhardness value of the sample.

## 3. Results and Discussion

### 3.1. The Effect of Process Parameters on the Surface Morphology

#### 3.1.1. The Effect of Laser Frequency on the Surface Morphology

**Figure 2:**
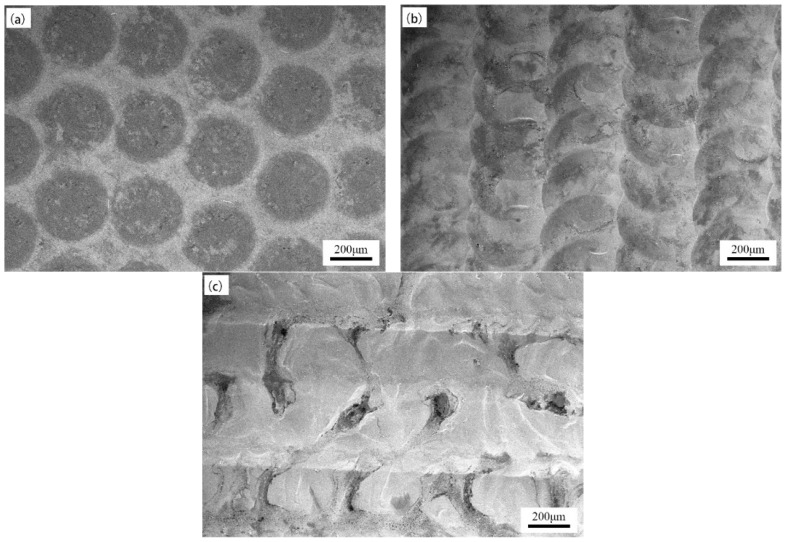
Surface morphology of samples under different laser frequencies: (**a**) 5 Hz, (**b**) 10 Hz, and (**c**) 15 Hz.

Figure 2 shows the surface morphology of the sample under different laser frequencies. Keeping the laser energy density at 132 J/mm^2^ and the laser pulse width at 10 ms, many circular remelting points with a diameter of approximately 250 μm appeared on the surface of the sample when the laser frequency was 5 Hz (Figure 2a). Although these remelting points exhibited a good linear distribution, there was no overlap between adjacent points. Therefore, the remelted layer of the sample was incomplete. When the laser frequency increased to 10 Hz (Figure 2b), as the pulse frequency increased, the remelting points overlapped and exhibited a fish scale-like structure. The overlap area between remelting points accounted for about 50% of the entire point. When the laser frequency continued to increase to 15 Hz (Figure 2c), the surface of the sample exhibited a ridge-like structure accompanied by obvious pits, and there were banded step protrusions between adjacent columns. The contour of the circular impact point basically disappeared.

The changes in surface morphology above-mentioned may be caused by the following reasons. When the laser frequency increases, the time interval between adjacent laser pulses will decrease. When the laser frequency is 5 Hz, a larger pulse interval time will reduce the number of remelting points produced per unit time. The melting point cannot fully cover the laser feed path, so the surface of the sample cannot obtain a complete remelted layer. When the laser frequency increases to 10 Hz, the number of impact points per unit time is moderate, which can effectively cover the scanning path of the device and ensure a certain proportion of stacking. When the laser frequency is 15 Hz, the interval between laser pulses is very short, and the overlapping degree of remelting points further increases. In addition, too short time an interval cannot meet the cooling requirements for remelting points. Therefore, when the remelting point overlaps under this parameter, the partially cooled remelting point will experience significant material splashing, resulting in the surface morphology shown in Figure 2c.

#### 3.1.2. The Effect of Laser Pulse Width on the Surface Morphology

**Figure 3:**
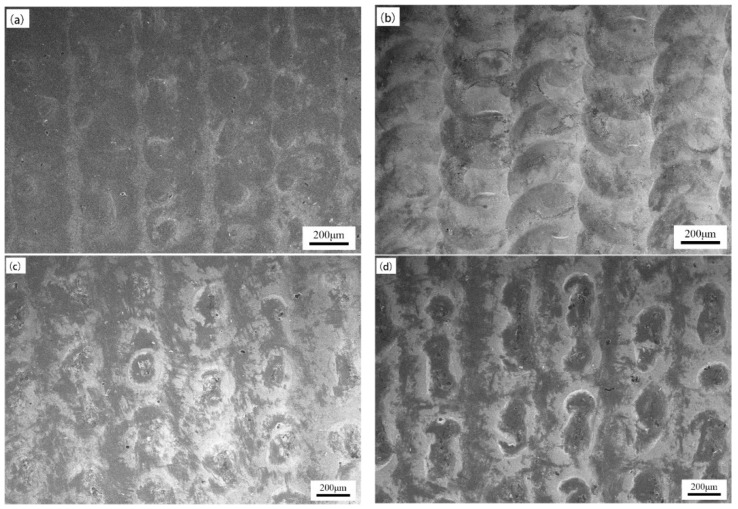
Surface morphology of the samples under different laser pulse widths: (**a**) 7.5 ms, (**b**) 10 ms, (**c**) 12.5 ms, and (**d**) 15 ms.

Figure 3 shows the surface morphology of the sample when the laser energy density is 132.69 J/mm^2^ and the laser pulse frequency is 10 Hz, and the laser pulse width parameter is changed separately. As shown in the figure, when the pulse width was 7.5 ms (Figure 3a), the area of the circular impact points formed by laser shock was small, the overlap between the impact points was low, and there was a significant banded omission area between adjacent rows. This may be due to the smaller pulse width at this time and the shorter laser irradiation time for a single impact point, resulting in insufficient impact intensity. When the pulse width was 12.5 ms and 15 ms (Figure 3c,d), the surface morphology of the sample was more disordered, and there were deep impact melting pits locally. The reason for this phenomenon may be due to the high impact strength caused by the larger pulse width at this time, and the splashing of the molten pool at the impact point.

#### 3.1.3. The Effect of Laser Energy Density on the Surface Morphology

**Figure 4:**
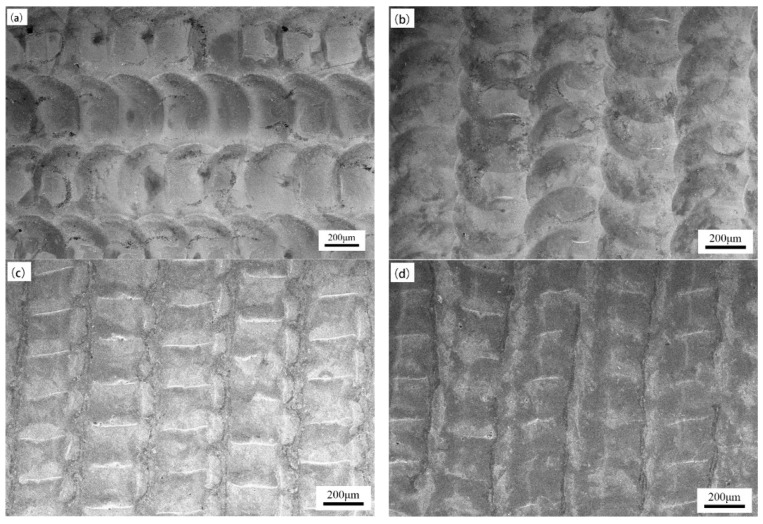
Surface morphology of samples under different energy densities: (**a**) 99.52 J/mm^2^, (**b**) 132.69 J/mm^2^, (**c**) 165.87 J/mm^2^, and (**d**) 199.04 J/mm^2^.

Figure 4 shows the surface morphology of the sample with the pulse frequency of 10 Hz and the pulse width of 10 ms, and the laser energy density was changed separately. Comparing Figure 4a–d, it can be seen that as the energy density increased from 99.52 J/mm^2^ to 132.69 J/mm^2^, the contour of the circular remelting points on the surface gradually deepened, and the boundaries between the impact points became clearer. When the energy density increased from 132.69 J/mm^2^ to 165.87 J/mm^2^, the contour of the remelting point became blurred. There were obvious bamboo-shaped protrusions between the impact points on the same scanning line, while there were more obvious strip-shaped protrusions between the adjacent two scanning lines. When the energy density increased to 199.04 J/mm^2^, the contour of the circular impact point basically disappeared, the width of the bamboo-shaped protrusions between the impact points became shorter, and the ribbon protrusions further increased. The reason for this phenomenon may be that when the energy density is low, the sample surface is less affected by the laser, so the boundary of the impact point is unclear. As the energy density gradually increased, the impact intensity of the laser gradually increased, resulting in a clear contour of the impact point. When the energy density was too high, the molten pool area at the impact point further increased. Interference occurred between two adjacent molten pools on the same scanning line, resulting in bamboo-shaped protrusions.

### 3.2. The Effect of Process Parameters on the Microhardness

#### 3.2.1. The Effect of Laser Frequency on the Microhardness

**Figure 5:**
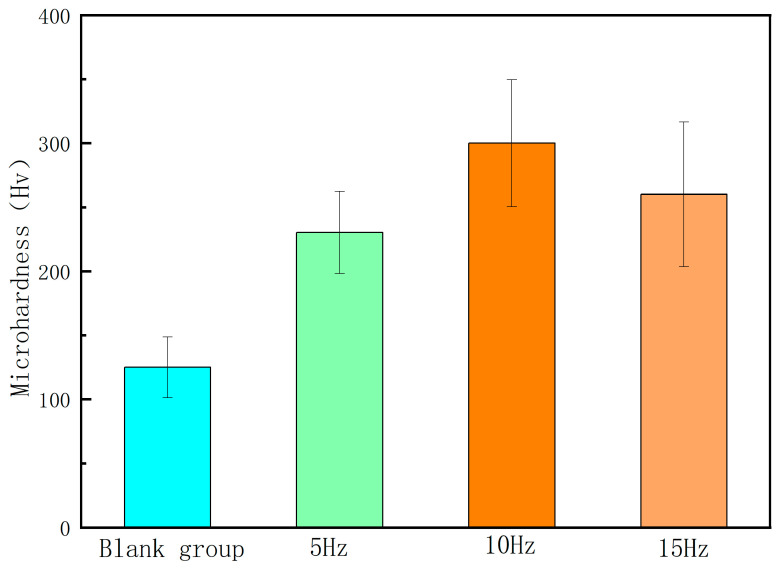
Microhardness of the samples at different laser frequencies.

Figure 5 shows the microhardness of the sample at different laser frequencies. From the figure, it can be seen that the microhardness value of the sample surface before laser remelting was relatively low, with an average value of about 125 Hv. With the gradual increase in laser frequency, the microhardness value of the sample showed a significant improvement and exhibited a trend of first increasing and then decreasing. When the laser frequency was 10 Hz, the average microhardness of the sample reached 300 Hv. Based on the analysis of the surface morphology of the sample, it can be concluded that the change in microhardness of the sample was mainly caused by the influence of laser frequency on the integrity of the remelted layer. When the laser frequency is low, the number of remelting points on the surface of the sample is rare, and a complete remelting layer is not formed, resulting in lower microhardness. When the laser frequency is too high, the partially cooled remelting points are excessively stacked, and serious splashing occurs on the surface of the sample during processing. This significantly increases the number of structural defects such as pores and inclusions in the remelted layer, resulting in a decrease in the microhardness value of the sample.

#### 3.2.2. The Effect of Laser Pulse Width on the Microhardness

**Figure 6:**
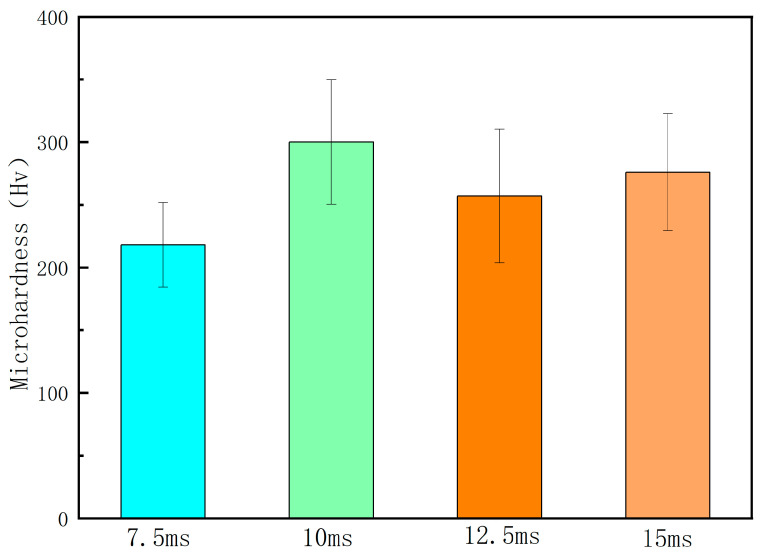
Microhardness of the samples at different laser pulse widths.

Figure 6 shows the microhardness of the samples at different laser pulse widths. From the figure, it can be seen that as the pulse width gradually increased, there was a slight fluctuation in the microhardness of the sample. When the pulse width increased from 7.5 ms to 10 ms, the microhardness of the sample increased from 218 Hv to 300 Hv. When the pulse width was 12.5 ms, there was a brief decrease in the microhardness of the sample, and the microhardness value was 257 Hv. As the pulse width continued to increase, there was a slight increase in the microhardness of the sample, with the microhardness value increasing to 276 Hv. This phenomenon is because as the pulse width gradually increases, the duration of the laser’s effect on the melting point also gradually increases. The change in remelting duration can increase the depth of the laser melt pool, which is beneficial for obtaining a better remelting layer thickness on the surface of the sample. Therefore, as the laser pulse width increased from 7.5 ms to 10 ms, the microhardness of the sample gradually increased. When the laser pulse width is increased to 12.5 ms, excessive laser energy input will cause the melting point temperature to be too high, resulting in boiling of the melt pool and serious material gasification and splashing phenomena. This increases the number of structural defects such as pores and inclusions in the remelted layer, reduces the uniformity of the remelted layer, and is therefore not conducive to improving the microhardness of the sample. When the laser pulse width continues to increase to 15 ms, although excessive laser energy input at this time can still cause serious gasification and splashing of the material, the depth of the molten pool also increases with the increase in the pulse width. A deeper melt pool can protect the deep remelted layer from the effects of surface material gasification and splashing, and the bottom of the remelted layer can obtain a more uniform microstructure. Therefore, there was a slight increase in the microhardness of the sample.

#### 3.2.3. The Effect of Laser Energy Density on the Microhardness

**Figure 7:**
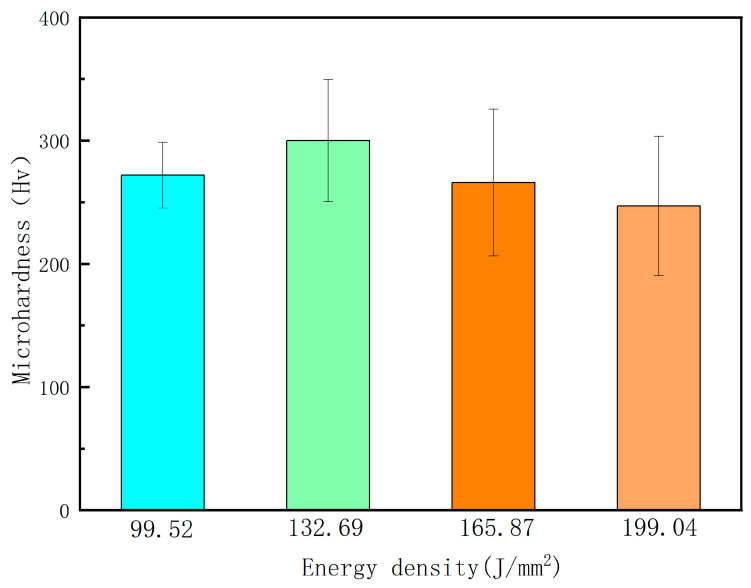
Microhardness of the samples at different energy densities.

Figure 7 shows the microhardness of the samples at different energy densities. According to the data in the figure, as the laser energy density gradually increased, the microhardness of the sample showed a trend of first increasing and then slowly decreasing. When the energy densities of the samples were 99.52 J/mm^2^, 132.69 J/mm^2^, 165.84 J/mm^2^, and 199.04 J/mm^2^, the corresponding microhardness values of the samples were 272 Hv, 300 Hv, 266 Hv, and 247 Hv, respectively. The maximum variation in microhardness between adjacent parameters was 34. The above-mentioned changes in microhardness of the sample were mainly related to the damage caused by laser energy density to the material. When the laser energy density exceeds the damage threshold of the sample, the laser has the ability to remelt the sample. The higher the laser energy density, the higher the instantaneous temperature rise obtained on the material surface. When the laser energy density increased from 99.52 J/mm^2^ to 132.69 J/mm^2^, the highest temperature value obtained on the material surface also increased. This not only enabled the cooled sample to obtain better tissue stress, but also helped to enhance the thickness of the remelted layer. Therefore, the microhardness of the sample surface showed an increasing trend. As the laser energy density continued to increase, the excessively high laser energy density could instantly cause gasification on the material surface. The gasification of materials not only increased the number of structural defects in the remelted layer, but also reduced the thickness of the remelted layer. Therefore, as the laser energy density further increased, the microhardness of the sample surface showed a decreasing trend.

### 3.3. The Effect of Process Parameters on the Surface Roughness

#### 3.3.1. The Effect of Laser Frequency on the Surface Roughness

**Figure 8:**
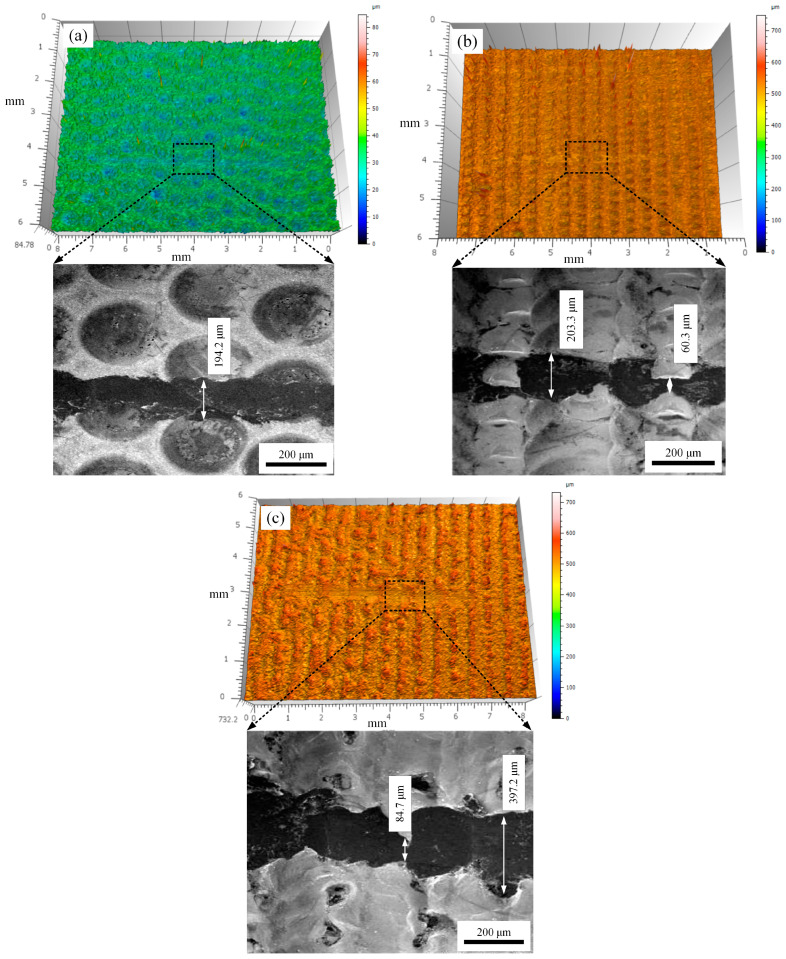
Three-dimensional morphology and SEM images of the sample wear mark under different laser frequencies: (**a**) 5 Hz, (**b**) 10 Hz, (**c**) 15 Hz.

Figure 8 shows the three-dimensional morphology and SEM images of the sample wear mark under different laser frequencies. From the figure, we can clearly see that as the pulse frequency increased, the sample surface changed from a uniform honeycomb-like structure to a furrow-like structure. When the pulse frequency is 15 Hz, although the sample surface still maintains a furrow-like main structure, its structural uniformity and consistency decrease.

By comparing the wear marks in the figure, it can be seen that when the laser frequency is 5 Hz, the width of the wear mark on the sample surface is relatively uniform, with a maximum width of about 194.2 μm. Under this parameter, the morphology of the wear mark conforms to the characteristics of adhesive wear, without obvious plow-like scratches and flaky detachment. Therefore, it can be inferred that the main wear form of the sample is adhesive wear. When the laser frequency is 10 Hz and 15 Hz, there is obvious flaky detachment of the remelted layer in the wear marks, and the consistency of the width decreases, with corresponding wear mark width differences reaching 140 and 312.5 μm, respectively. Therefore, the wear form of the samples under this parameter is mainly fatigue wear. The main reasons for the differences in wear forms mentioned above are the concentration of surface stress and tissue defects in the materials. After laser treatment, the rapid cooling of the remelting point will generate a large amount of stress concentration on the surface of the material. When the laser frequency is low, the number of remelting points on the surface of the sample is small and the coverage is incomplete. Therefore, the sample still exhibits the wear characteristics of copper. When the laser frequency increases, the remelted layer completely covers the surface of the sample and there is a large amount of stress concentration. Severe stress concentration leads to the appearance of microcracks inside the remelted layer, and the periodic reciprocating friction forces cause the microcracks to propagate and extend, ultimately resulting in the detachment of the remelted layer. In addition, when the frequency is too high, the time and position interval between adjacent remelting points further narrows, causing excessive overlap of remelting points. When the partially solidified remelting point is subjected to a second remelting, material splashing will occur on the surface of the sample, leading to an increase in the number of structural defects such as pores and slag inclusions in the remelted layer. This further exacerbates the growth of microcracks and the possibility of detachment of remelted layers.

**Figure 9:**
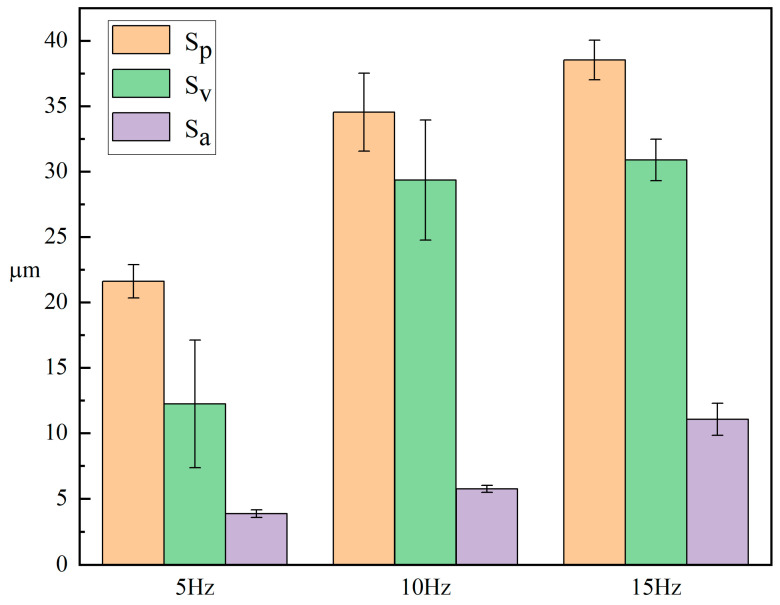
Surface roughness of samples under different laser frequencies.

Figure 9 shows the surface roughness of the samples at different laser frequencies. The data in the figure shows that the surface roughness of the sample increases with the increase of pulse frequency. When the frequency is 15 Hz, the surface roughness reaches its maximum, approximately 11.050 μm. However, the difference between the peak and valley values of rough structures does not increase with the roughness. This phenomenon is mainly caused by the following two reasons. On the one hand, as the laser frequency increases, the number of remelting points on the surface of the sample gradually increases, leading to an increase in its surface roughness. On the other hand, When the pulse frequency is low, there is no material flow between adjacent remelting points, resulting in a large difference between their surface peaks and valleys. As the laser frequency increases, the stacking of adjacent remelting points has a peak shaving and valley filling effect, so the difference between their peaks and valleys does not further increase. When the frequency is too high, the molten pool undergoes multiple laser remelting during the cooling process, leading to increased material gasification and splashing losses, resulting in a slight increase in the difference between the surface peaks and valleys of the sample. So, during the process of increasing pulse frequency, although the surface roughness of the sample increases, the difference between its surface valleys and peaks decreases compared to 5 Hz.

#### 3.3.2. The Effect of Laser Pulse Width on the Surface Roughness

**Figure 10:**
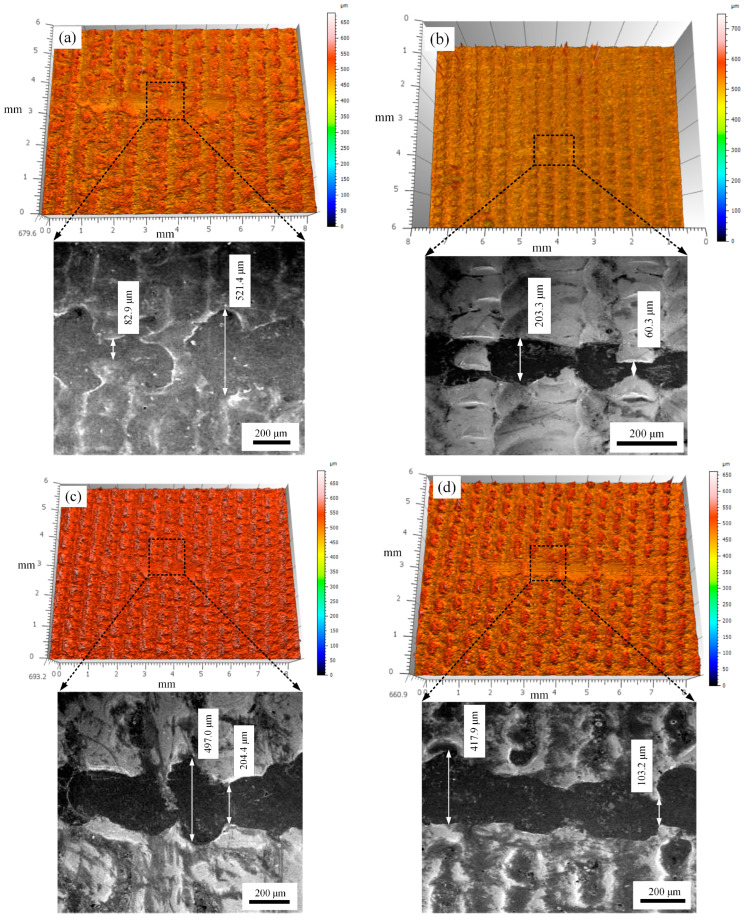
Three-dimensional morphology and SEM images of the sample wear mark under different laser pulse widths: (**a**) 7.5 ms, (**b**) 10 ms, (**c**) 12.5 ms, and (**d**) 15 ms.

**Figure 11:**
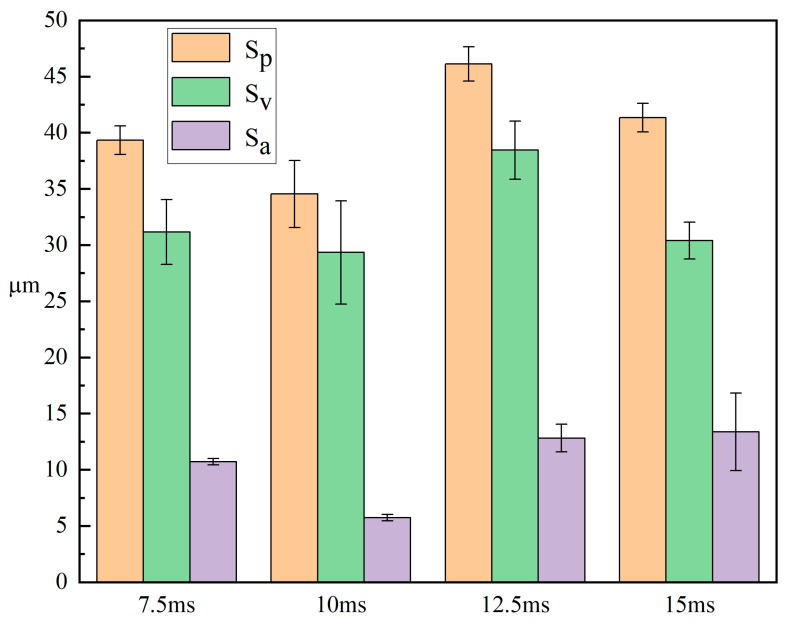
Surface roughness of samples under different pulse widths.

Figure 10 shows the three-dimensional morphology and SEM images of the sample wear mark under different laser pulse widths. From the figure, it can be seen that the surface of the samples exhibited a furrow-like microstructure. With the exception of the sample with the pulse width of 10 ms, the integrity of the furrow-like microstructure on the surface of all other samples showed different levels of damage. The surface roughness of the samples under different pulse widths is shown in Figure 11. From the data in the figure, it can be seen that when the pulse width increased from 7.5 ms to 15 ms, the surface roughness of the sample was 10.709 μm, 5.735 μm, 12.810 μm, and 13.370 μm, respectively. The overall surface roughness of the sample showed a trend of decreasing first and then increasing. This result is consistent with the SEM and three-dimensional morphology display of the sample. The reason for the above changes in surface roughness of the sample may be due to the variation in the duration of laser action on the sample. When the pulse width is low, the laser remelting time on the sample is short, and the insufficient temperature rise at the remelting point leads to poor fluidity of the melt pool. The material at the remelting point cannot fill the previous remelting point well, resulting in higher roughness. When the pulse width is too large, the laser remelting time on the surface of the sample is longer, and the material temperature rise is too high, resulting in the splashing phenomenon, which affects the surface roughness of the sample.

The SEM images of the sample wear mark showed that the average wear width of the sample fluctuated as the pulse width increased from 7.5 ms to 15 ms. Among them, when the laser pulse width was 12.5 ms, the wear on the sample surface was the highest, with an average width of approximately 345.2 μm. When the pulse width was 10 ms, the wear on the surface of the sample was the smallest, with an average width of about 132 μm. After wear, the remelted layer showed different levels of flaky detachment at the edges and bottom of the wear marks. Based on this, it can be determined that the main form of wear of the above samples was fatigue wear. The main reason for this result is related to the surface stress concentration and structural defect changes of the sample after laser remelting. It is worth noting that when the pulse width was 15 ms, the enhancement of the wear resistance of the sample may also have been related to the depth of remelting. When the laser pulse width is too large, although increasing the duration of laser remelting can cause material splashing at the remelting point and generate more structural defects such as pores and slag inclusions, the depth of the remelting pool at the remelting point also further increases. A better depth of the molten pool prevents the formation of pores and slag inclusions in the remelted layer, resulting in a better microstructure. Therefore, when the pulse width was 15 ms, the wear resistance of the sample was enhanced.

#### 3.3.3. The Effect of Laser Energy Density on the Surface Roughness

**Figure 12:**
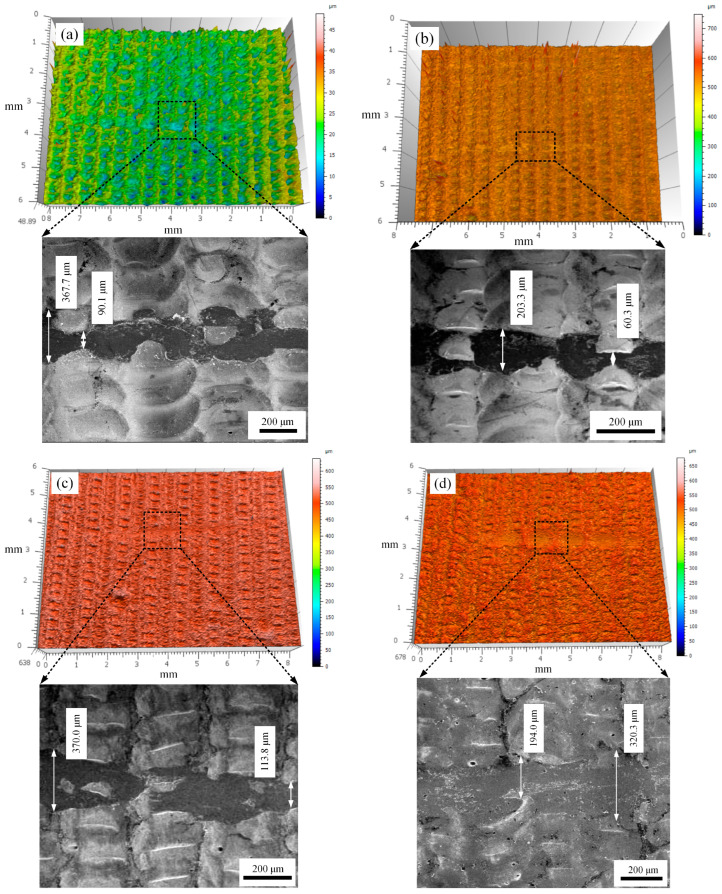
Three-dimensional morphology and SEM images of the sample wear mark under different energy densities: (**a**) 99.52 J/mm^2^, (**b**) 132.69 J/mm^2^, (**c**) 165.87 J/mm^2^, and (**d**) 199.04 J/mm^2^.

**Figure 13:**
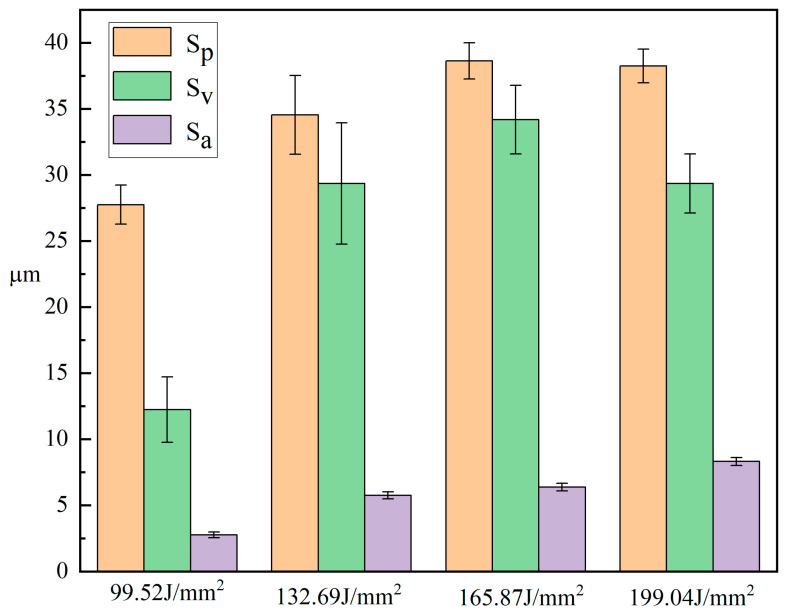
Surface roughness of samples under different energy densities.

Figure 12 shows the three-dimensional morphology and SEM after wear of samples under different energy densities. From the three-dimensional morphology of the sample, it can be seen that the overall surface structure of the sample fluctuated relatively smoothly and exhibited good uniformity. Figure 13 shows the surface roughness under different energy densities. According to the data in the figure, when the energy density increased from 99.52 J/mm^2^ to 165.87 J/mm^2^, the surface roughness of the sample increased from 2.752 μm to 8.301 μm. Although the surface roughness values showed a gradually increasing trend, their overall variation amplitude was weaker than that under laser frequency and pulse width. This result indicates that compared to laser frequency and pulse width, the influence of laser current parameters on the surface quality of the sample is relatively small.

The SEM images of the sample wear mark showed that with the continuous increase in the energy density, the average width of wear marks on the sample was 228.7 μm, 132 μm, 241.9 μm, and 257.15 μm, respectively. The overall wear resistance of the sample showed a trend of increasing first and then decreasing. By comparing and analyzing the wear characteristics of the worn position, it can be concluded that the wear form of the sample was still mainly fatigue wear. From the previous analysis, it can be seen that the main reasons for this phenomenon were stress concentration and structural defects in the remelted layer. As is well-known, when the laser damage threshold remains constant, the higher the laser energy density, the greater the laser damage to the material. When the laser energy density is low, the thickness of the remelted layer is thin and cannot cope with long-term wear tests, resulting in a larger width of the wear marks. When the laser energy density exceeds the damage threshold too much, the sample will experience severe gasification and material splashing, leading to an increase in defects in the remelted layer structure and a decrease in wear resistance.

In addition, compared to the influence of laser frequency and laser pulse width parameters on the wear resistance, the fluctuation amplitude of the sample wear width is smaller when the laser energy density changes. This may be related to the surface quality and structural consistency of the remelted layer. By comparing the surface morphology and 3D structural morphology of the samples under different parameters, it can be concluded that the remelted layer on the surface of the samples has good surface quality under different laser energy densities. The enhancement in surface quality and microstructure consistency is beneficial for alleviating stress concentration in the remelted layer, thereby improving the wear resistance of the remelted layer.

## 4. Conclusions

This article took the laser frequency, pulse width, and energy density as the research objects to test and characterize the microstructure, surface roughness, and wear resistance of the copper surface after laser remelting strengthening. The relevant results indicate that the laser frequency, pulse width, and energy density can directly affect the surface quality of the sample, but the influence of the laser frequency and pulse width on the surface quality is more prominent. When the frequency was 10 Hz, the pulse width was 10 ms, and the energy density was 132.69 J/mm^2^, the remelting points on the surface of the sample exhibited a regular fish scale-like structure. The overlapping area of impact points accounted for about 50% of the impact point area, and the surface quality and wear resistance of the sample were superior to the other parameters.

## Data Availability

Data is contained within the article or supplementary material.
